# A Randomized Feasibility/Acceptability Trial of Acceptance and Commitment Therapy for People with HIV Who Drink at Unhealthy Levels

**DOI:** 10.1007/s10461-025-04990-7

**Published:** 2025-12-29

**Authors:** Sarah E. Woolf-King, Michelle R. Dalton, Madison Firkey, Alan Sheinfil, Veronica Bucci, Brianna Estrada, Jeremy Ramos, Judith A. Hahn, Jonathan Bricker, Brooks Gump, Kestutis G. Bendinskas, Stephen A. Maisto

**Affiliations:** 1 Department of Psychology, Syracuse University, Syracuse, NY 13244 USA; 2 Department of Family and Community Medicine, St. Louis University, St. Louis, MO USA; 3 Department of Psychiatry and Behavioral Sciences, SUNY Upstate Medical University, Syracuse, NY USA; 4 School of Psychology and Counseling, Farleigh Dickinson University, Teaneck, NJ USA; 5 Department of Psychiatry, College of Medicine, University of Illinois Chicago, Chicago, IL USA; 6 Behavioral Medicine Program, Massachusetts General Hospital, Boston, MA USA; 7 Department of Psychiatry, University of Rochester, Rochester, NY USA; 8 Department of Medicine, University of California, San Francisco, CA USA; 9 Fred Hutchinson Cancer Research Center, Seattle, WA USA; 10 Department of Psychology, University of Washington, Seattle, WA USA; 11 Department of Public Health, Syracuse University, Syracuse, NY USA; 12 Department of Chemistry, State University of New York at Oswego, Oswego, NY USA

**Keywords:** Alcohol, HIV, Acceptance, Transdiagnostic

## Abstract

Unhealthy alcohol use is prevalent among people with HIV (PWH) and associated with significant health-related consequences. We describe here a randomized feasibility/acceptability trial of a brief, telephone-delivered transdiagnostic acceptance and commitment therapy (ACT) intervention for PWH who drink at unhealthy levels. This pilot trail was designed to match the procedures of a planned definitive RCT that will compare the ACT intervention to a brief alcohol intervention (BI). We randomly assigned PWH and unhealthy alcohol use 1:1 to either the ACT or the BI intervention and collected self-report and biomarker data post-treatment and again at 3-, 6-, and 12-months. The primary objectives of this pilot trial were to assess feasibility of recruitment, retention, and intervention delivery, and acceptability of intervention content. We also collected preliminary primary (alcohol use) and secondary (symptoms of anxiety, depression, and experiential avoidance) outcome data to examine general patterns of results at a purely descriptive level. In total, 192 participants were screened, 117 of whom were eligible, and 49 of whom were randomized to either the ACT (*n* = 24) or BI (*n* = 25) condition. Results provided evidence of feasibility and acceptability indicating that a definitive trial should proceed. Preliminary outcome data also suggested that ACT is a promising treatment for unhealthy alcohol use and co-morbid symptoms of depression, anxiety, and experiential avoidance. A definitive trial is currently underway and will determine the comparative efficacy of ACT versus BI for unhealthy alcohol use for PWH.

Unhealthy alcohol use—which encompasses a range of consumption from drinking that exceeds recommended guidelines to alcohol use disorder (AUD) [1, 2]—is prevalent among people with HIV (PWH). Between 27 and 40% of PWH report drinking at unhealthy levels [3–5] which is associated with a range of health-related consequences, including mortality [6], lack of viral suppression [7, 8], less ART utilization [8, 9], and sub-optimal adherence to ART [10].

Randomized clinical trials (RCTs) of alcohol interventions for PWH have had mixed success. There have been two meta-analyses [11, 12] and several narrative reviews [13–15] of this literature published over the last decade. Of the trials conducted in the U.S. with adult samples of PWH, only three trials [16–18] have produced significant effects on alcohol-related outcomes that were different from the control group at 12-month follow-up. One of the three trials that found significant treatment effects was Chander et al.’s [16] brief intervention based on Project Treat [19]. This intervention is a two-session (20 min each) + two booster call (5–10 min) CBT-based treatment that significantly reduced past 90-day drinking frequency compared to usual care, with treatment effects that were maintained at 12-month follow-up. The brief format, low training burden, and significant treatment effects have made Chander et al.’s [16] intervention widely adopted in treating unhealthy alcohol use among PWH. However, the lack of treatment effects on drinking quantity, and the exclusive focus on alcohol use, indicate there may be potential for improvement in the implementation of brief alcohol interventions for PWH.

One hypothesized reason for the mixed success of alcohol interventions in the HIV literature is an increasingly recognized syndemic of co-occurring unhealthy alcohol use and other mental health-related problems among PWH [20, 21]. Indeed, up to 63% of PWH meet criteria for a co-occurring substance use and psychiatric disorder [22, 23], with depression and anxiety being the most common psychiatric co-morbidities [23, 24]. The high level of psychological and behavioral comorbidities among PWH necessitates a shift towards examining transdiagnostic approaches that target core psychological processes that underlie multiple mental health and substance-related problems [14].

## Experiential Avoidance as a Transdiagnostic Mechanism

One transdiagnostic mechanism that is particularly relevant to alcohol and other substance use is experiential avoidance (i.e., repeated, and maladaptive attempts to escape, avoid, or modify unwanted thoughts, bodily sensations, emotions, and/or urges) [25]. The inability and/or unwillingness to tolerate these uncomfortable internal experiences contributes to habitually avoidant behavior that often takes the form of alcohol and other substance use [25]. Indeed, experiential avoidance has been found to be a transdiagnostic factor in the etiology and maintenance of multiple psychiatric disorders or symptoms [25–28]. Transdiagnostic interventions that target experiential avoidance, such as acceptance and commitment therapy (ACT), increase psychological acceptance and flexibility by building skills that alter the function of unwanted internal experiences rather than attempting to change their content or frequency. The development of mindfulness skills, clarification of core personal values, and engagement in values-based committed action that comprises ACT, decreases experiential avoidance and increases psychological flexibility.

## Evidence for ACT

A 2020 review [29] of 20 meta-analyses on 100 controlled effect sizes found that ACT was efficacious across a broad range of intervention targets, including depression, anxiety disorders, chronic pain, and stress. To our knowledge, only one meta-analysis has been published that concentrated exclusively on ACT for substance use [30]. All the trials focused on drug use (e.g., nicotine, amphetamines, opiates) and used an active treatment control condition, with ACT superior at post-treatment (g = 0.29) and follow-up (g = 0.43) timepoints. The positive effects of ACT increased over time, or at least deteriorated less quickly, compared to other active treatment conditions, a “sleeper effect” that is hypothesized to be rooted in change related to the core mechanism of action (decreased experiential avoidance), resulting in continued improvement in outcomes over time [30].

The literature on ACT for alcohol use is less well-developed with only three preliminary studies published thus far [31–34]. Given the limited literature on ACT for alcohol use, the brief ACT for substance use interventions provide an excellent template for further development of ACT-based alcohol interventions. The smoking cessations studies are particularly relevant given the innovative telephone, web-based, and smartphone-based platforms used in those studies, and the high comorbid psychiatric and medical conditions of the populations included in the ACT for smoking cessation trials. Of the five RCTs of ACT for smoking cessation that Lee et al. [30] analyzed, three were conducted by Bricker et al. [35–37], who have continued to publish in this area since that time [38, 39]. Bricker et al.’s TALK Intervention [36], a 5-session, telephone-delivered ACT intervention for smoking cessation, was recently compared to a standard CBT-based intervention (matched for length and intensity) with promising results [36]. The availability of ACT in this brief, manualized, telephone-delivered format makes it an ideal framework from which to adapt a treatment capable of addressing the multiple needs of PWH who drink at unhealthy levels.

## Specific Objectives of this Feasibility/Acceptability Trial

We adapted Bricker et al.’s ACT-based smoking cessation intervention (the TALK Intervention) into an intervention tailored to PWH who drink at unhealthy levels (the ACCEPT Intervention). A detailed description of the adaptation process that we followed has been previously published [40, 41]. We describe here the randomized feasibility/acceptability trial we subsequently conducted to inform a future full-scale comparative efficacy trial in which we will directly compare the ACCEPT Intervention to Chander et al.’s [16] brief alcohol intervention. We report here on the procedures, challenges encountered, and feasibility/acceptability data relevant to informing a follow-up, fully powered comparative efficacy trial. We also present primary and secondary outcomes at a purely descriptive level in line with the shift in the National Institutes of Health’s (NIH’s) stance on the appropriate use of pilot trials and several influential publications describing misuse of pilot trial data [42–44]. Consistent these recommendations, we did not conduct formal inferential statistics for hypothesis testing related to the primary outcomes, nor did we use the pilot trial for estimation of effect sizes for the full-scale trial.

## Methods

### Overview

This randomized feasibility/acceptability trial was designed to match the procedures of a planned full-scale RCT such that participants were randomized 1:1 to one of two study conditions and to a study interventionist within that condition. All participants attended a baseline, post-treatment, 3-month, and 6-month study visit. Given budget constraints we initially did not have a 12-month study visit but received additional funding to add one halfway through recruitment for the pilot trial. Both intervention conditions involved six phone calls for equivalence in interventionist contact. The trial was registered on clinicaltrials.gov (NCT03974061), all procedures were approved by the Institutional Review Board (IRB) at Syracuse University, and our reporting is consistent with the CONSORT extension for randomized pilot and feasibility trials [45].

A priori, we established that the following feasibility and acceptability outcomes would indicate readiness to proceed with the full-scale trial: (1) we expected that completion rates of the ACCEPT intervention, and retention of research participants, would match or exceed those reported by Chander et al.’s CBT-based brief alcohol intervention (BI) and Bricker et al.’s TALK intervention – i.e., ≥  ~ 75%, (2) we expected that participants would endorse the ACCEPT intervention as “acceptable”, defined as at least > 5 on a standardized 12-item measure of treatment acceptability that uses a 7-point Likert-type scale. This score would indicate that participants at least “often” felt the intervention was acceptable to them (3) we expected that our training of study interventionists would be sufficient for implementation of the ACCEPT and BI interventions with at least 90% fidelity to our treatment manuals, and (4) at a purely descriptive level, the primary and secondary outcomes that will be the focus of the full-scale trial would demonstrate an overall pattern consistent with a decline in symptoms of unhealthy alcohol use, symptoms of depression, and symptoms of anxiety in both conditions, as well as a decline in symptoms of experiential avoidance in the ACCEPT condition only.

## Inclusion/Exclusion Criteria

Inclusion criteria required participants to be adult (18+), PWH, on ART who reported unhealthy alcohol use in the past month on the alcohol use disorder identification test-consumption (AUDIT-C; ≥ 4 men; ≥ 3 women). Participants were excluded if they scored > 20 on the patient health questionnaire (PHQ-9), indicating severe symptoms of depression, > 15 on the General Anxiety Disorder (GAD-7) questionnaire, indicating severe symptoms of anxiety, or > 11 on the AUDIT-C, indicating high risk for severe AUD. Participants were also excluded if they indicated symptoms of acute psychosis on the brief symptom inventory (BSI) that would interfere with their ability to consent and/or participate in the intervention, and if they were unable to read at an 8th grade level (the reading level at which the workbooks for each intervention were written). Finally, mid-way through the study we implemented an inclusion criterion that required participants to identify a physical address where they could be reached if emergency services had to be called due to acute emotional distress. This was added after a participant endorsed suicidal ideation that required a welfare check, prompting our IRB to require this change.

## Sample Size Considerations

The target sample size was determined by balancing the need for an adequate number of participants to provide data supporting feasibility and acceptability, while also staying within the allocated budget and time constraints associated with a developmental pilot trial. As noted above, the sample size was not intended to provide sufficient power for inferential statistics, nor sufficient power to provide effect size estimates to inform sample size calculations for the full-scale trial. We also note that randomization is less effective at balancing groups with small sample sizes [46], and thus the randomization reported here is focused on piloting of procedures. With these considerations in mind, our original target enrollment sample was *N* = 74.

## Procedures

### Recruitment and Enrollment

This pilot trial was originally designed to recruit participants in-person from a single, local HIV clinic during a regularly scheduled medical visit, and recruitment began in Fall of 2019. Four months into the trial, in March 2020, when the COVID-19 pandemic shuttered all in-person research in the state of New York, we transitioned the study to completely remote participation. As such, our recruitment efforts shifted online and widened to include, at first, the state of New York, and eventually the entire United States (U.S.). In our first attempt at online recruitment, we created a database of 1165 HIV-and substance use-related service organizations, sending over 6000 emails to advertise the study. When this proved insufficient, we successfully partnered with TrialFacts, a company that specializes in online recruitment for clinical trials. This company developed media advertisements specifically for our study and used advanced online recruitment algorithms targeting multiple online sources. Participants who viewed an advertisement for the study on one of these platforms were directed to a study landing page that pre-screened them for eligibility. Eligible participants could then schedule a full screening appointment with our study staff using a calendar embedded in the landing page. Recruitment for the trial continued through November 2021, and the last study visit was completed in November 2022.

### Baseline and Follow-up Study Visits

Remote study visits occurred via telephone, with the option for videoconference according to participant preference (no participant preferred the latter). Prior to the baseline visit, participants were mailed a study “welcome kit” that included a hard copy of the informed consent, incentive accrual handout, instructions for self-collection of biospecimens (feasibility/acceptability data on self-collection of biomarkers for alcohol and stress have been previously published [47]), introductions to study staff, and a handout listing national mental health resources. A research assistant (RA) called participants at their scheduled visit time, re-screened for eligibility, and then sent a link to an electronic informed consent that was reviewed in detail. After consent, self-reported measures were interviewer-administered and entered directly into a REDCap database, the secure data management web application that housed all study data.

Participants were randomized 1:1 to either the ACT or BI condition, and an interventionist within that condition, using the randomization module in REDCap. Participants were then asked to self-collect biospecimens and mail their samples back to the lab PO Box within one-week of their study visit, using a pre-paid priority mail envelope. Follow-up study visits occurred immediately post-treatment, and again at 3-, 6-, and (for some participants) 12-months post-baseline. These visits were identical with the exception of consent and randomization in the baseline, and the acceptability questionnaire in the post-treatment session only. Outcome assessors were blinded to treatment condition. Participants were paid $50 for their participation in each study visit, $5 for each intervention session, and a $50 bonus for successfully completing all study visits.

### Intervention Content

Table 1 provides a comparison of intervention content from the ACT and BI Conditions. Both interventions were delivered by Masters-level interventionists enrolled in a PhD program in Clinical Psychology, all of whom had foundational training in basic counseling skills. The study had between 3–4 interventionists at a time, all of whom were trained in both interventions. Training for the ACCEPT intervention involved reading ACT Made Simple [48], The Happiness Trap [49], and the treatment manual developed for this study [40]. Subsequently, interventionists were required to role-play each session with Principal Investigator (SWK), who is a licensed clinical psychologist and who received training in ACT via both formal course work and via an in-person training with Dr. Bricker and his team. ACT interventionists therefore received approximately 10 h of supervised role-play training and were considered ready for implementation if their role-play sessions covered at least 90% of the content in the ACT Fidelity Monitoring Checklists that were also used in the fidelity monitoring described below.

**Table 1:** Comparison of intervention content in the acceptance and commitment therapy (ACT) and brief intervention (BI) conditions

	Call #1	Call #2	Call #3	Call #4	Call #5	Call #6
ACT	Education: Alcohol and HIV	Education: Values vs Goals	Education: Creative Hopelessness	Education: Unhooking	Education: Self-care	Education: Course of behavior change
	Skill: Self-monitoring	Skill: Three Breaths	Skill: Pause-Allow-Take a Breath (PAT)	Skill: Thoughts on a stream	Skill: List of things to care for oneself	Skill: List of favorite tools from intervention
	Metaphor/Activity: Car Journey	Metaphor/Activity: Celebration of Life	Metaphor/Activity: Quicksand	Metaphor: Thoughts as a radio in background	Metaphor: Puppy	Metaphor: Quicksand, Thoughts as Weather, Values as Compass
	Action plan: Goal for practice of self-monitoring	Action plan: Goal for practice of Three Breaths and alcohol use reduction	Action plan: Goal for practice of PAT and alcohol use reduction	Action plan: Goal for practice of Thoughts on a Stream and alcohol use reduction	Action plan: Goal for practice of self-care and alcohol use reduction	Action plan: Final goal for alcohol use and practice of skills
	Length: 45–60 min	Length: 30–40 min	Length: 30–40 min	Length: 30–40 min	Length: 30–40 min	Length: 30–40 min
BI	Education: Harmful effects of alcohol use; Low risk drinking levels	Check-in on drinking agreement	Remind participant of their next full intervention session	Exercises: Strategies for drinking reduction goals: get support, drink refusal, alternative activities, engagement in health behaviors, reward behavior change	Check-in on drinking agreement	Remind participant of their next study visit
	Exercises: Risks and reasons for drinking and ways to cut down	Problem-solve challenges			Problem-solve challenges	
	Drinking agreement: goal for alcohol reduction and self-monitoring	Reinforce alcohol use reduction		Drinking agreement: goal for alcohol reduction, self-monitoring and practice of drinking reduction strategies	Reinforce alcohol use reduction	
	Length: 45–60 min	Length: 10–15 min	Length: < 5 min	Length: 30–45 min	Length: 10–15 min	Length: 10–15 min

Training for the BI condition was led by the co-principal investigator (SAM), also a licensed clinical psychologist with decades of clinical and research experience supervising brief alcohol interventions. Interventionists read the treatment manual our team adapted from Chander et al.’s work and role-played each session with SAM until proficiency was achieved. The supervised role-play training time was approximately 5 h for the BI intervention.

Once the trial started, all study interventionists had one hour of weekly group supervision with SAM and SWK to discuss implementation, monitor cross-contamination, and discuss any issues that arose with either. All treatment sessions were audio recorded (with consent) for review during group supervision and for subsequent fidelity monitoring.

#### Content of BI Sessions

Participants randomized to the BI condition received a 2 session + 2 booster call, standard BI, adapted for telephone-delivery, from Chander et al.’s BI [16]. The CBT-oriented manualized intervention included the creation of a drinking agreement, self-monitoring via drinking diary cards, discussion of risky moods/situation, strategies for managing these moods/situations, and identification of alternative activities to replace alcohol use. In order to standardize the number of treatment contacts across conditions, participants received a full (45–60 min) BI session in week 1, a 5–10 min booster call in week 2, a < 5 min reminder call in week 3, a second full (30–45 min) BI session in week 4, a 5–10 min booster call in week 5, and a < 5 min reminder call in week 6. The booster sessions were focused on progress towards drinking goals, and the reminder calls were simply to remind the participant of their next regularly scheduled intervention session or study appointment.

#### Content of ACT Sessions

Participants randomized to the ACCEPT intervention received the manualized, 6-session, telephone-delivered ACT intervention adapted by our team [40]. Sessions were approximately 30-min long, except for the first session, which was 45–60 min. Session #1 provided an overview of the ACT model plus education about alcohol (e.g., standard drinks, safe drinking limits), HIV, and the association between them (e.g., alcohol use and adherence). Sessions 2–4 each covered a different ACT-related topic—Values (Session #2), Acceptance (Session #3), Cognitive Defusion (Session #4), and Self-compassion (Session #5). Each session was structured to include psychoeducation about the ACT topic, an ACT-based metaphor (e.g., Thoughts as Radio Playing in the Background), an ACT-based skill (e.g., Thoughts on a Moving Stream), and a committed action plan for practice of ACT skills and reduction in drinking behavior. The sessions began with a review of progress on the prior weeks’ committed action plan (for both drinking and practice of ACT skills) and ended with a new plan tailored to the participant’s readiness to change and to the specific ACT skill for the week. The participant was encouraged to reflect on the ways in which their action plan was related to the core values identified in Session #2, ensuring that behavior change goals were values-based.The final call (Session #6) included a summary of all the ACT skills learned throughout the intervention, psychoeducation on the variable course of behavior change, and a final committed action plan.

### Measures

#### Feasibility

Feasibility was assessed by determining the number of participants recruited and retained, the number of intervention sessions completed, and the number of treatment “completers”—defined a priori as completion of at least 4 sessions for both treatments.

#### Acceptability

We used a standardized 12-item measure of acceptability that asked participants to indicate how “often” they felt the intervention was important to them, helped them work on their problems, and helped them make meaningful changes [50]. Questions were answered using a 7-point Likert-type scale ranging from 1 = never to 7 = always.

#### Treatment fidelity

Standardized checklists were used to determine fidelity to treatment manuals in both conditions and 20% of treatment sessions were randomly selected and double coded by RAs trained in the fidelity monitoring protocol. Specifically, treatment “completers” (i.e., participants who completed at least 4 sessions) were eligible for fidelity monitoring and of the completers, 20% of the completed sessions were randomly selected without replacement for fidelity monitoring. Four trained research assistants were randomly assigned to coder teams comprised of a primary and secondary coder. Upon completion of fidelity monitoring, 10% of the calls were randomly selected to be cross-checked by the project coordinator for consistency. Each coder was individually trained on the fidelity monitoring checklists adapted to match each treatment manual. For example, the fidelity monitoring checklist for session #1 of the ACT intervention included 23 yes/no items that mapped onto the manualized treatment content (e.g., “reviewed car journey metaphor” and “reviewed safe drinking recommendations”). Prior to official onset of fidelity monitoring, each coder practiced with two participants and was required to code all six sessions with the fidelity monitoring checklists, which were reviewed for accuracy by the PIs and project coordinator.

### Preliminary Outcomes

#### Alcohol Use

Past six-week alcohol use was measured via the timeline follow-back (TLFB) [51]. We calculated number of drinks per drinking day and number of drinking days over the previous six-weeks as primary alcohol-related outcomes.

#### Symptoms of Depression and Anxiety

Symptoms of depression were measured with the patient health questionnaire (PHQ-9) [52], a brief, standardized measure of depression severity, and symptoms of anxiety were measured with the general anxiety disorder (GAD-7) [53], a brief, standardized measure of anxiety severity.

#### Symptoms of Experiential Avoidance

Symptoms of experiential avoidance were measured with the brief experiential avoidance questionnaire (BEAQ) [54], a 15-item measure that assesses six domains of experiential avoidance (e.g., avoidance of feelings, avoidance of taking action).

## Results

### Participants

Given the disruptions caused by the COVID-19 pandemic described previously, we were able to enroll and randomize 66% (*N* = 49) of our target sample size. Participants were 92% male, 47% Black (39% White, 10% mixed race, and 4% another race or ethnicity), were, on average, 53.65 years old (*SD* = 9.29) and had been living with HIV for ~ 20 years (*M* = 19.51, *SD* = 10.41). The majority (65%) of participants in the sample made less than $39,000/year, and 69% identified as gay or queer (see Table 2).

**Table 2:** Participant demographics

Variables	ACT (*n* = 24)		BI (*n* = 25)		Overall (*N* = 49)	
	*M*	*SD*	*M*	*SD*	*M*	*SD*
Age	53.67	9.18	53.64	9.59	53.65	9.29
Years since HIV diagnosis	20.25	11.37	18.80	9.58	19.51	10.41

	*n*	*%*	*n*	*%*	*n*	*%*
*Sex assigned at birth*						
Male	22	91.67	23	92.00	45	91.84
Female	1	4.17	2	8.00	3	6.12
Decline to answer	1	4.17	0	0.00	1	2.04
*Sexual orientation*						
Heterosexual/straight	5	20.83	4	16.00	9	18.37
Homosexual/gay/queer	15	62.50	19	76.00	34	69.39
Bisexual	3	12.50	2	8.00	5	10.20
Decline to answer	1	4.17	0	0.00	1	2.04
*Race*						
Black or African American	13	54.17	10	40.00	23	46.94
Caucasian/white	9	37.50	10	40.00	19	38.78
Mixed Race	1	4.17	4	16.00	5	10.20
Another ethnicity	1	4.17	1	4.00	2	4.08
*Income*						
Less than $10,000	4	16.67	7	28.00	11	22.45
$10,000–19,999	4	16.67	2	8.00	6	12.24
$20,000–29,999	4	16.67	5	20.00	9	18.37
$30,000–39,999	4	16.67	2	8.00	6	12.24
$40,000–49,999	0	0.00	0	0.00	0	0.00
$50,000–59,999	1	4.17	2	8.00	3	6.12
$60,000–69,999	0	0.00	1	4.00	1	2.04
$70,000–79,999	1	4.17	1	4.00	2	4.08
$80,000–89,999	1	4.17	0	0.00	1	2.04
$90,000–99,999	0	0.00	1	4.00	1	2.04
$100,000 or more	2	8.33	2	8.00	4	8.16
Decline to answer	3	12.50	2	8.00	5	10.20
*Region of residence*						
Northeast	12	50.00	18	43.00	30	61.22
Midwest	1	4.17	0	0.00	1	2.04
South	10	41.67	6	24.00	16	32.65
West	1	4.17	1	4.00	2	4.08

### Feasibility

**Fig. 1:**
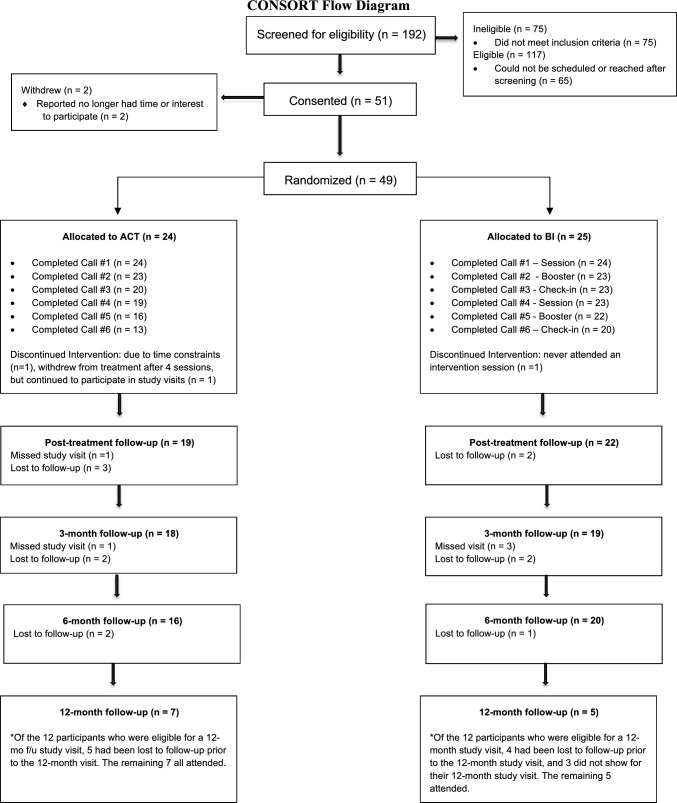
Consort diagram

A total of 192 participants were screened, 117 of whom were eligible, 51 of whom were enrolled and consented, and 49 of whom were randomized to the ACT (*n* = 24) or BI (*n* = 25) intervention condition (see Fig. 1). Most of the participants (82%) were considered treatment “completers” (i.e., completed at least four treatment sessions). The majority (84%) also completed a post-treatment follow-up visit, 75% completed their 3-month study visit, and 73% completed their 6-month study visit. As noted above, given that the 12-month study visit was added midway through the study, only 24 participants were eligible for it, and of those, 9 had been lost to follow-up prior to the 12-month visit. Of the remaining 15 participants, 12 completed, with an overall retention of 50% (*n* = 12) at 12-months.

### Acceptability

As shown in Table 3, mean scores on the acceptability survey were > 5 on all items for both the ACT and BI conditions. Participants reported that they “very often” felt that what they were working on was important to them: (*M*_ACT_ = 6.11, *SD*_ACT_ = 1.15; *M*_BI_ = 6.29, *SD*_BI_ = 0.96) and “very often” believed that the way they were working on their problems in the intervention would “lead to positive changes in (their) life” (*M*_ACT_ = 6.16, *SD*_ACT_ = 1.17; *M*_BI_ = 5.63, *SD*_BI_ = 1.36).

**Table 3:** Post-treatment acceptability outcomes

		ACT (N = 19)		BI (N = 22)	
Item	Description	M	SD	M	SD
1	I agree with the program about the things I will need to do to help improve my situation	5.32	1.38	5.64	1.47
2	What I am doing in these sessions gives me new ways of looking at my problem	5.42	1.26	5.36	1.53
3	My interactions with the technology- based program are positive	6.16	0.90	6.32	1.04
4	The program does not understand what I am trying to accomplish in these sessions	1.37	0.76	2.27	1.72
5	I am confident in the technology-based program's ability to help me	5.84	1.61	5.36	1.73
6	The program and I are working towards my goals	6.16	0.96	6.27	0.94
7	I feel appreciated in this treatment	6.32	1.11	6.5	0.81
8	I am working on what is important to me in these sessions	6.11	1.15	6.29	0.96
9	I trust what the program is doing for me	6.11	1.33	5.95	1.25
10	The program has different ideas than me about what my problems are	2.95	1.81	3.86	2.05
11	The program has helped me establish a good understanding of changes that would be good for me	5.84	1.30	5.57	1.69
12	I believe the way we are working on my problems will lead to positive changes in my life	6.16	1.17	5.63	1.36

### Treatment Fidelity

Each ACT intervention call ranged from 18–40 min, with call 1 being the longest (*M* = 40.2, *SD* = 9.65) and call 6 being the shortest (*M* = 18.68, *SD* = 23.72). For the BI, the two primary intervention sessions lasted on average 55 (*SD* = 16) and 36 (*SD* = 20) minutes respectively (calls 1 and 4), the two booster calls lasted on average 9–10 min each (calls 2 and 5), and the two reminder phone calls added to ensure equity in interventionist contact (calls 3 and 6) lasted < 5 min each.

Fidelity monitoring data indicated that our training and supervision procedures were successful. All the randomly selected sessions were double coded with high percent agreement (average percent agreement across selected sessions = 95%). The average adherence score for the ACT condition was 96% and the average adherence score for the BI condition was 98%, indicating high treatment fidelity, and providing objective evidence that, with the training procedures, treatment manuals, and clinical supervision we developed and tested in this pilot trial, the interventionists successfully and accurately implemented both intervention conditions.

### Preliminary Outcomes

Means and standard deviations for primary and secondary outcomes across all time points for both conditions are presented in Table 4.

**Table 4:** Preliminary outcome data

	Baseline				Post-treatment				3-months				6- months				12- months			
	ACT (*N* = 24)		BI (*N* = 25)		ACT (*N* = 19)		BI (*N* = 22)		ACT (*N* = 18)		BI (*N* = 19)		ACT (*N* = 16)		BI (*N* = 20)		ACT (*N* = 7)		BI (*N* = 5)	
	M	(SD)	M	(SD)	M	(SD)	M	(SD)	M	(SD)	M	(SD)	M	(SD)	M	(SD)	M	(SD)	M	(SD)
# of drinking days	26.21	(13.68)	21.2	(12.97)	17.21	(16.26)	12.95	(12.93)	17.35	(16.39)	12.11	(13.05)	19	(18.66)	9.47	(12.64)	12.86	(12.07)	20.83	(16.06)
# of drinks/drinking day	4.55	(2.58)	3.99	(2.18)	4.28	(3.74)	2.85	(1.90)	3.38	(3.47)	2.69	(1.79)	3.2	(3.45)	2.84	(2.44)	2.70	(2.59)	4.09	(1.85)
Depression (PHQ-9)	14.5	(4.02)	16.76	(4.32)	13.37	(3.30)	15.23	(4.24)	12.67	(3.50)	14.89	(3.46)	12.38	(3.59)	13.85	(3.84)	12.29	(4.23)	14.80	(1.92)
Anxiety (GAD-7)	11.96	(4.16)	13.04	(4.03)	10.42	(2.65)	12.73	(4.70)	9.67	(2.87)	12.05	(3.44)	8.6	(1.80)	11.5	(3.99)	8.14	(1.21)	12.00	(1.73)
Experiential avoidance (BEAQ)	47.83	(13.95)	50.48	(14.01)	45.42	(12.49)	49.6	(12.40)	44.24	(17.05)	52.72	(14.20)	42	(15.92)	49.65	(14.37)	43.86	(13.74)	58.60	(11.50)

#### Alcohol-related Outcomes (see Figs. 2, 3)

The overall pattern of results indicated a decline in number of drinking days for both conditions. The average number of drinking days at baseline was 26.21 (*SD* = 13.86) in the ACT condition and 21.20 (*SD* = 12.97) in the BI condition. Post-treatment, number of drinking days had declined by 34.34% in the ACT condition and 38.91% in the BI condition. These reductions were largely maintained by the 6-month follow-up in both conditions. At 12-month follow-up drinking frequency in the BI condition increased to near baseline levels (*M* = 20.83, *SD* = 16.06), with an overall 1.75% reduction from baseline. The ACT condition showed evidence of a continued pattern of decline in drinking frequency (*M* = 12.86, *SD* = 12.07) with an overall 50.93% reduction from baseline.

Average number of drinks per drinking day showed a similar pattern. The average number of drinks per drinking day at baseline was 6.24 (*SD* = 3.06) in the ACT condition and 5.48 (*SD* = 2.63) in the BI condition. The overall pattern of results indicated a decline in number of drinks per drinking day for both conditions. At the post-treatment follow-up, drinks per drinking day had declined by 19.16% in the BI condition and remained largely the same in the ACT condition. By 6-month follow-up, drinks per drinking day had declined by 48.18% in the BI condition and 48.72% in the ACT condition. Similar to number of drinking days, the trajectory for drinks per drinking day continued downward for ACT (*M* = 2.70, *SD* = 2.59) at the 12-month follow-up, ending with an overall 56.73% reduction from baseline. Conversely, the number of drinks per drinking day began to increase in the BI condition (*M* = 4.09, *SD* = 1.85) at 12-month follow-up with an overall 25.37% decrease from baseline.

**Fig. 2:**
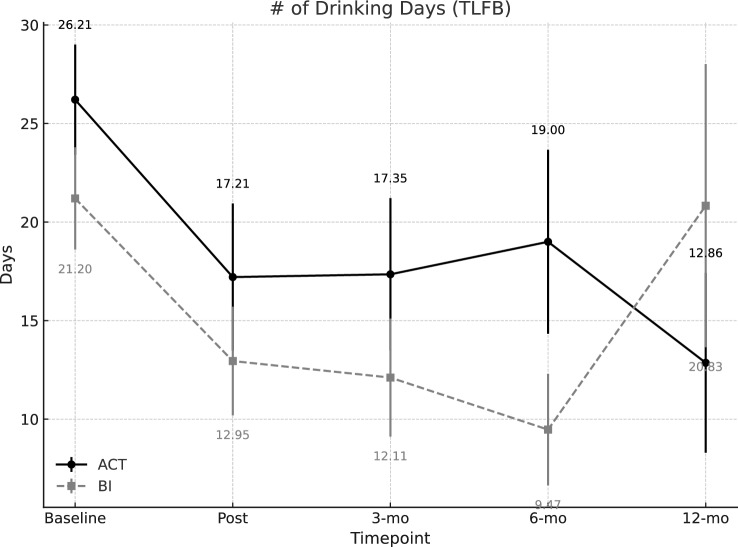
Number of drinking days

**Fig. 3:**
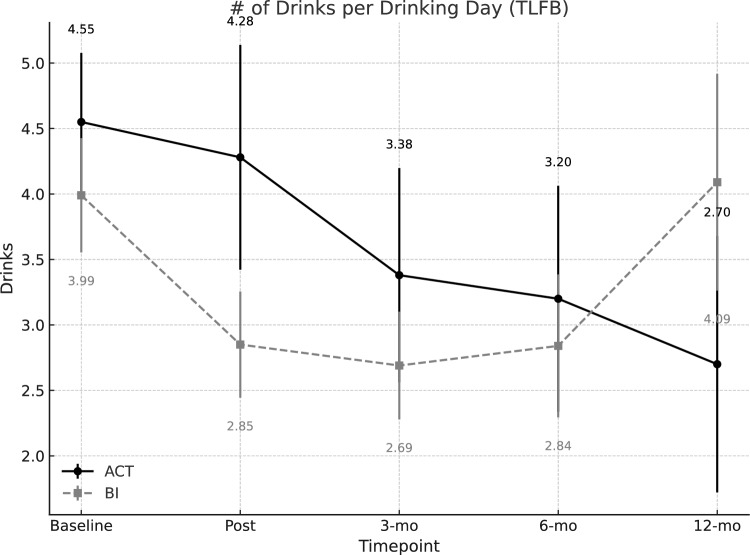
Number of drinks per drinking day

#### Symptoms of Anxiety and Depression (see Figs. 4, 5)

The overall pattern of results indicated a decline in symptoms of depression and anxiety for both conditions. The average score on the PHQ-9 at baseline was 14.50 (*SD* = 4.02) in the ACT condition and 16.76 (*SD* = 4.32) in the BI condition. At the post-treatment follow-up, symptoms of depression had declined by 7.79% in the ACT condition and 9.13% in the BI condition. By 6-month follow-up, symptoms of depression had declined by 14.62% in the ACT condition and 17.36% in the BI condition. The trajectory of depressive symptoms continued downward for ACT (*M* = 12.29, *SD* = 4.23) at the 12-month follow-up, ending with an overall 14.24% reduction from baseline. Conversely, the number of depressive symptoms began to rebound in the BI condition (*M* = 14.80, *SD* = 1.92) at 12-month follow-up, with an overall 11.69% decrease from baseline.

**Fig. 4:**
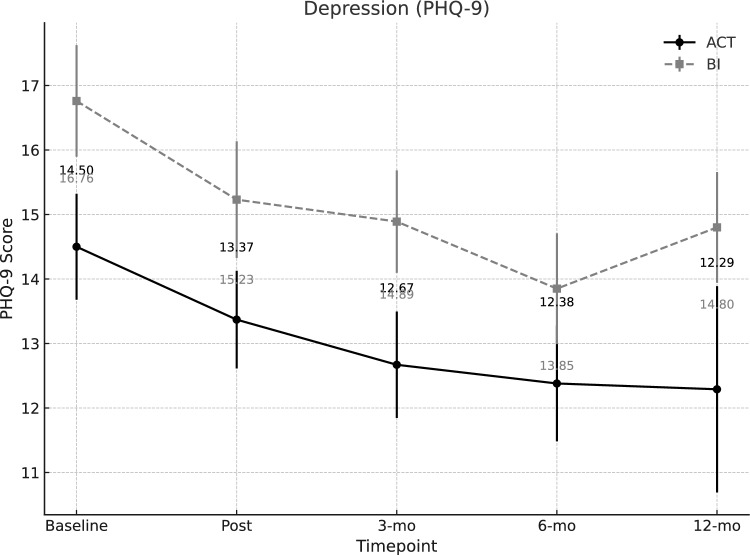
Symptoms of depression

The results for symptoms of anxiety were similar. There was a steady decrease in symptoms of anxiety for both conditions. The average score on the GAD-7 at baseline was 11.96 (*SD* = 4.16) in the ACT condition and 13.04 (*SD* = 4.03) in the BI condition. At the post-treatment follow-up, symptoms of anxiety had decreased by 12.88% in the ACT condition and 2.38% in the BI condition. These reductions were maintained at 6-month follow-up, with symptoms of anxiety having declined by 28.09% from baseline in the ACT condition and 11.81% from baseline in the BI condition. Again, symptoms of anxiety continued to decrease for the ACT condition (*M* = 8.14, *SD* = 1.21) with an overall 31.94% decline from baseline. For the BI condition, symptoms of anxiety began to rebound (*M* = 12.00, *SD* = 1.73), ending with an overall 7.98% decline from baseline.

**Fig. 5:**
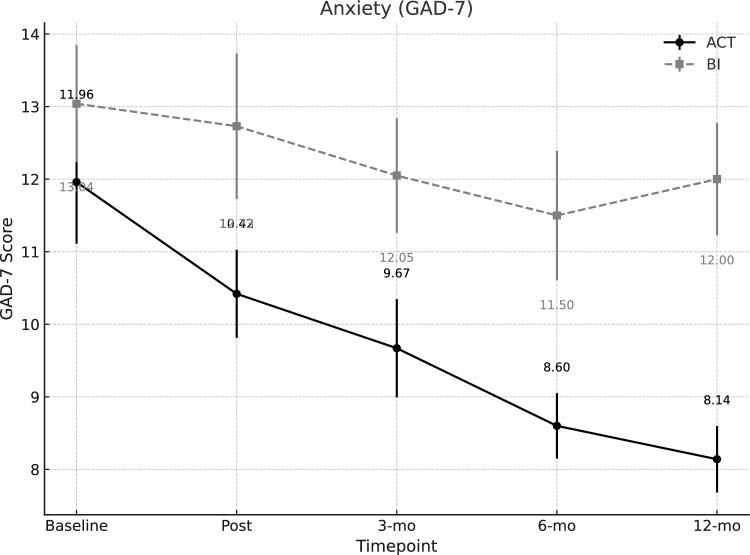
Symptoms of Anxiety

#### Symptoms of Experiential Avoidance (see Fig. 6)

The overall pattern of results for symptoms of experiential avoidance suggested a steady decline in the ACT condition, with a more variable trajectory for the BI condition. At baseline, the average score on the BEAQ was 47.83 (*SD* = 13.95) in the ACT condition and 50.48 (*SD* = 14.01) in the BI condition. At post-treatment follow-up, symptoms of experiential avoidance had decreased by 5.04% in the ACT condition and 1.74% in the BI condition. At 6-month follow-up, the decrease from baseline was more pronounced in the ACT condition (12.19%) compared to the BI condition (1.64%). The ACT condition finished with an overall 8.3% decline from baseline to 12-months, while the BI condition finished with an overall 16.09% *increase* in symptoms of experiential avoidance from baseline.

**Fig. 6:**
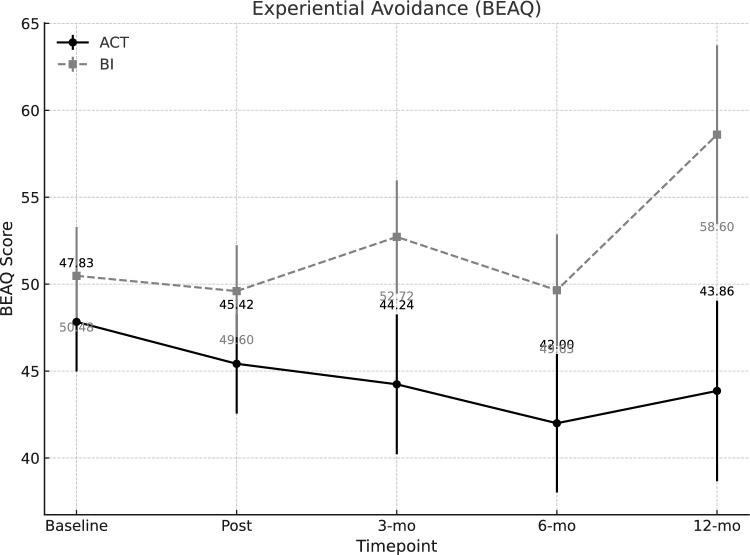
Symptoms of experiential avoidance

## Discussion

This randomized pilot trial provided evidence for feasibility and acceptability in support of the execution of a full-scale comparative efficacy trial, which is currently underway (NCT06648629). Our expectations related to feasibility (retention, number of treatment completers), acceptability, and fidelity monitoring were largely supported. The preliminary outcome data suggested that ACCEPT is a promising brief, telephone-delivered treatment for unhealthy alcohol use and co-morbid symptoms of depression and anxiety for PWH. Preliminary outcome data also indicate that ACCEPT shows potential for enacting change in experiential avoidance, an important transdiagnostic mechanism of change, underscoring its potential and utility to treat other medical populations with similar needs. This feasibility/acceptability trial provided several insights that informed the proposal for the full-scale RCT, all of which improved its design.

### Interpretation of Pilot Trial Objectives and Applications to Definitive Trial

Feasibility data indicated that we could successfully recruit and retain PWH who meet the inclusion/exclusion criteria for the planned definitive trial. The trial and error we encountered recruiting PWH remotely, and across the entire U.S., informed the methods we will use in the definitive trial. Our most fruitful recruitment method was our work with Trialfacts, which allowed us to access far more potential participants than reaching out personally to HIV and substance use service organizations. We thus allocated a significant proportion of the budget to develop online advertising and use an RCT recruitment company for the duration of the definitive trial, which should improve the efficiency of our recruitment methods. Additionally, using an RCT recruitment company allows for participants to schedule screening appointments directly with study staff immediately after clicking on an advertisement by directing them to a study landing page. This will ensure that preliminary screenings for eligibility are quickly translated into baseline study visits, reducing the gap between the number of participants screened and the number enrolled.

Also related to feasibility of recruitment, women were underrecruited in this pilot trial, and thus we proposed additional methods for the full-scale RCT. First, the upfront investment in online recruitment allowed for the development of materials that are more directly targeted to women, and the algorithms used in online recruiting can be flexibly applied to ensure these materials reach this demographic. If these strategies fail to recruit an adequate number of women, we also plan to partner with a large, ongoing cohort of women with HIV—the MACS/WIHS combined cohort study—to more directly advertise the study. Indeed, we added an investigator to the team that is directly involved with MACS/WIHS, which will allow us to access this collaborative research effort that involves 14 study sites in different regions of the U.S.

Feasibility of participant retention to 6-months was also demonstrated in this pilot trial, although with some room for improvement. The proliferation of literature on the conduct of virtual/remote trials in the wake of the COVID-19 pandemic [55, 56], and the designation of the definitive trial as virtual from the outset, allowed us to enhance our retention procedures. For example, we will use electronic e-gift card payments that can be digitally delivered to participants, via text, immediately upon completion of study visits. In the pilot trial, we used physical gift cards, and eventually money orders, that were mailed to participants, which caused several problems (e.g., delays in receipt of payment, lost cards) that may have affected retention. We will also add a small incentive to the definitive trial ($5 e-gift card) for mailing biospecimen kits back to the lab within one-week, which we hope will increase our ability to directly compare biospecimens to self-reported data at a given study visit.

The feasibility of retention to 12-months in this pilot trial was influenced by the addition of this study visit mid-way through the study. As noted above, we applied for and received, additional funding that allowed us to pilot procedures for a 1-year of follow-up, and to incorporate the self-collection of fingernails for the assessment of cortisol—an indicator of hypothalamic–pituitary–adrenal (HPA) axis activity in body’s response to stress. The full-scale trial is designed, and appropriately funded, to incorporate a 12-month study visit at the outset of the trial, and we accordingly enhanced our procedures to increase retention. For example, we will contact participants biweekly in between appointments and use an automated service to deliver these messages. In addition, we will send automated study reminders one-week and 24 h prior to their study visit. Further, participants will receive a $50 bonus e-gift card for completing all study visits through 12-months.

### Pilot Trial Limitations

There are several limitations that should be considered when interpreting the data we report here. First, the small number of participants who were assessed at 12-month follow-up make the outcome assessment data at this timepoint especially difficult to interpret. While we emphasize throughout our commitment to refrain from formal hypothesis testing and use only descriptive data to comment generally on an overall pattern of results, this overall pattern is inconclusive, and the change in the direction of the overall pattern at the 12-month timepoint is unreliable. The full-scale trial will allow for the definitive determination of treatment effects, and any changes in their magnitude or direction over time.

Second, by having all of the study interventionists trained in both ACT and BI, there was some potential for crossover effects and bias in treatment delivery. For example, it was clear the study was designed to test the superiority of ACT, and interventionists may have unintentionally delivered the ACT treatment with more enthusiasm or conviction. Although neither were noted during fidelity monitoring, and group supervision and review of recorded sessions protected against this, we made several changes to the full-scale trial to address this concern. There will be two interventionists per study condition, both of whom will only deliver and be supervised on *either* ACT or BI. In this way, participants will be randomly assigned to a treatment condition, and to one of two interventionists within that condition who only deliver that specific treatment. Further, group supervision will occur separately and be led by two different licensed clinical psychologists—SWK for the ACT condition and SAM for the BI condition.

Third, because the recruitment procedures for the trial changed from a single HIV clinic in Central New York, to the whole state of New York, to the entire U.S., the sample was biased in terms of regional representation. Further, the sample was predominantly male, potentially limiting generalizability. The full-scale trial will address this by targeting the entire U.S. from the outset and monitoring regional representation as enrollment proceeds, as well as having a quota and enhanced recruitment strategies for women participants to ensure more balance in gender.

### Implications for Progression to Definitive Trial

The pilot trial described here provided essential feasibility and acceptability data that indicate a definitive trial should move forward. Indeed, this recently funded trial will determine the efficacy of ACCEPT compared to a standard BI. We expect superiority of the ACCEPT intervention in the full-scale trial after 6-months due to a “sleeper effect” reported in some ACT trials [30] where post-treatment effects are noted to not only be maintained, but to improve during follow-up timepoints. This is purportedly due to transformation in the core transdiagnosic mechanism of change—experiential avoidance. These effects may be enhanced by the telephone-delivery of the ACCEPT intervention, which has the potential to reach people who would otherwise decline such services due to shame or stigma. If our hypotheses are ultimately supported, the ACCEPT intervention will be the among the first evidence-based transdiagnostic interventions for alcohol use among PWH, which has broad implications for providers working in medical settings with high levels of alcohol use and other mental health co-morbidities.
